# Leaf nutritional composition and calcium speciation of a karst-endemic wild vegetable used by the indigenous people of Hainan Island, China

**DOI:** 10.3389/fnut.2026.1749876

**Published:** 2026-02-24

**Authors:** Ren-Fen Wang, Fang Wen, Ke Tan, Wen-Qian Xiang, Zhe Zhang, Yi-Gang Wei, Ming-Xun Ren, Ping-Ya Chen

**Affiliations:** 1 Guangxi Key Laboratory of Plant Conservation and Restoration Ecology in Karst Terrain, Guangxi Institute of Botany, Guangxi Zhuang Autonomous Region and Chinese Academy of Sciences, Guilin, China; 2 Hainan Institute of National Park & Key Laboratory for National Park Protection and Development of Hainan Province, Haikou, China; 3 International Joint Center for Terrestrial Biodiversity around South China Sea of Hainan Province, School of Ecology, Hainan University, Haikou, China; 4 School of Breeding and Multiplication (Sanya Institute of Breeding and Multiplication), Hainan University, Haikou, China; 5 Haikou Customs Technical Center, Haikou, China

**Keywords:** ethnobotany, Gesneriaceae, Hainan Island, *Primulina heterotricha*, traditional knowledge

## Abstract

*Primulina heterotricha*, a species endemic to Hainan Island, China, belongs to the Gesneriaceae family and is traditionally consumed as a wild vegetable by the Li ethnic group, the earliest inhabitants on the island. Despite its long history of use, systematic research on this species has been lacking. This study presents the first comprehensive analysis of the nutritional composition of *P. heterotricha*. The results reveal a high moisture content (95.4 g/100 g), and is rich in dietary fiber (2.95 g/100 g); it is characterized by low energy value (3.72 kcal/100 g) and low carbohydrate content (0.709 g/100 g). Notably, the species is characterized by exceptionally high calcium levels (289 mg/100 g), of which the predominant form is NaCl-Ca (133 mg/100 g), while other forms include H_2_O-Ca (67.8 mg/100 g), AIC-Ca (55.9 mg/100 g), HCl-Ca (15.3 mg/100 g), Res-Ca (8.80 mg/100 g), and HAC-Ca (8.60 mg/100 g). These findings not only confirm the high nutritional value of *P. heterotricha* as a calcium-rich wild vegetable resource but also underscore its potential applications in functional foods, the development of local specialty ingredients, and the preservation of the traditional dietary culture of the Li ethnic group. Moreover, this work provides a solid foundation for further research on its food industry applications and highlights the importance of documenting and safeguarding ethnobotanical knowledge in minority-inhabited regions of Hainan Island.

## 1 Introduction

Hainan Island, a tropical island in southern China, it forms part of the globally recognized Indo-Burma biodiversity hotspot and supports a rich diversity of wild vascular plants, including 516 endemic species (1–3). This unique biodiversity is closely associated with the island's complex topography and tropical monsoon climate, with the majority of endemic species concentrated in its central and southern mountainous regions (4, 5). Such plant diversity here provides local residents, especially indigenous ethnic minorities, with abundant plant resources and basic means of production.

The Li ethnic group, the indigenous inhabitants of Hainan Island, migrated from Guangdong and Guangxi before the Qin Dynasty (221–206 BC). They mainly live in the central and southern mountainous regions, including well-known high-altitude areas such as Wuzhi Mountain, Yinggeling, Limuling, Bawangling, and Yajiadaling (6). Over their long history in these mountains, the Li people incorporated the region's rich plant resources into daily life, production, and culture, developing unique traditions in the use of edible and medicinal plants (7). Over 80 species were traditionally consumed by the Li ethnic group (8, 9). Together with cultivated crops, these plants form a distinctive dietary structure of local people (10). Today, such wild vegetables—valued as “natural,” “distinctive,” and “health-promoting”—hold significant potential for further development and utilization.

*Primulina heterotricha* is a perennial herb endemic to Hainan (11). It primarily grows in valley forests and on streamside rocks within karst landscapes at elevations of around 430 m in central and southern Hainan (11–13). Traditionally, the whole plant has been used in folk medicine for treating kidney deficiency, bleeding, cough, and dampness (14). Among the Li ethnic group, tender leaves are harvested each spring (March–April) and prepared as soup (8, 15). Unlike other widely known wild vegetables in Hainan, such as *Crassocephalum crepidioides* and *Sauropus androgynus, P. heterotricha* is consumed only within a limited Li ethnic group and is rarely found in markets or restaurants (8). In addition to its dietary and medicinal uses, the species also possesses ornamental value owing to its attractive bluish-purple and light yellow flowers (16, 17).

The objectives of this study are to: (1) document traditional knowledge of *P. heterotricha* among the Li ethnic group; (2) quantify its nutritional composition; and (3) explore its potential for sustainable use and conservation, thereby providing a scientific basis for the development and utilization of this traditional wild edible vegetable, supporting regional socio-economic development, and contributing to biodiversity conservation in minority-inhabited areas.

## 2 Materials and methods

### 2.1 Ethnobotanical survey

In conducting this ethnobotanical study on *Primulina heterotricha*, we employed a multifaceted approach, including Google Scholar (https://scholar.google.com/), SpringerLink (https://link.springer.com/), PubMed (https://pubmed.ncbi.nlm.nih.gov/), Wiley Online Library (https://onlinelibrary.wiley.com/), and CNKI (https://www.cnki.net/). Additionally, we consulted sources, such as the Flora of China (18), Flora Hainanica (19), Inventory of Plant Species Diversity of Hainan (20), List of Species in Hainan (21), and Illustrated Handbook of Plants in Hainan (11) were also consulted to comprehensively review the current state of research concerning this plant. Our primary objective was to gather information on the traditional uses of *P. heterotricha*.

Within the Bawangling region of Hainan Province, we conducted ethnobotanical interviews focusing on *P. heterotricha* to delve into its local folk applications and utilization methods. A total of 53 informants were selected using the snowballing method, including experienced harvesters and elderly individuals familiar with the folk applications of *P. heterotricha*. These interviews were conducted using a semi-structured interview format, addressing aspects such as the local distribution areas, harvesting methods, processing techniques, and practical uses of *P. heterotricha* (22). All interviewees were informed of the purpose of the interviews and consent was obtained. In addition, we also recorded the whole process of *P. heterotricha* processing through the method of participatory observation.

### 2.2 Plant materials

**Figure 1:**
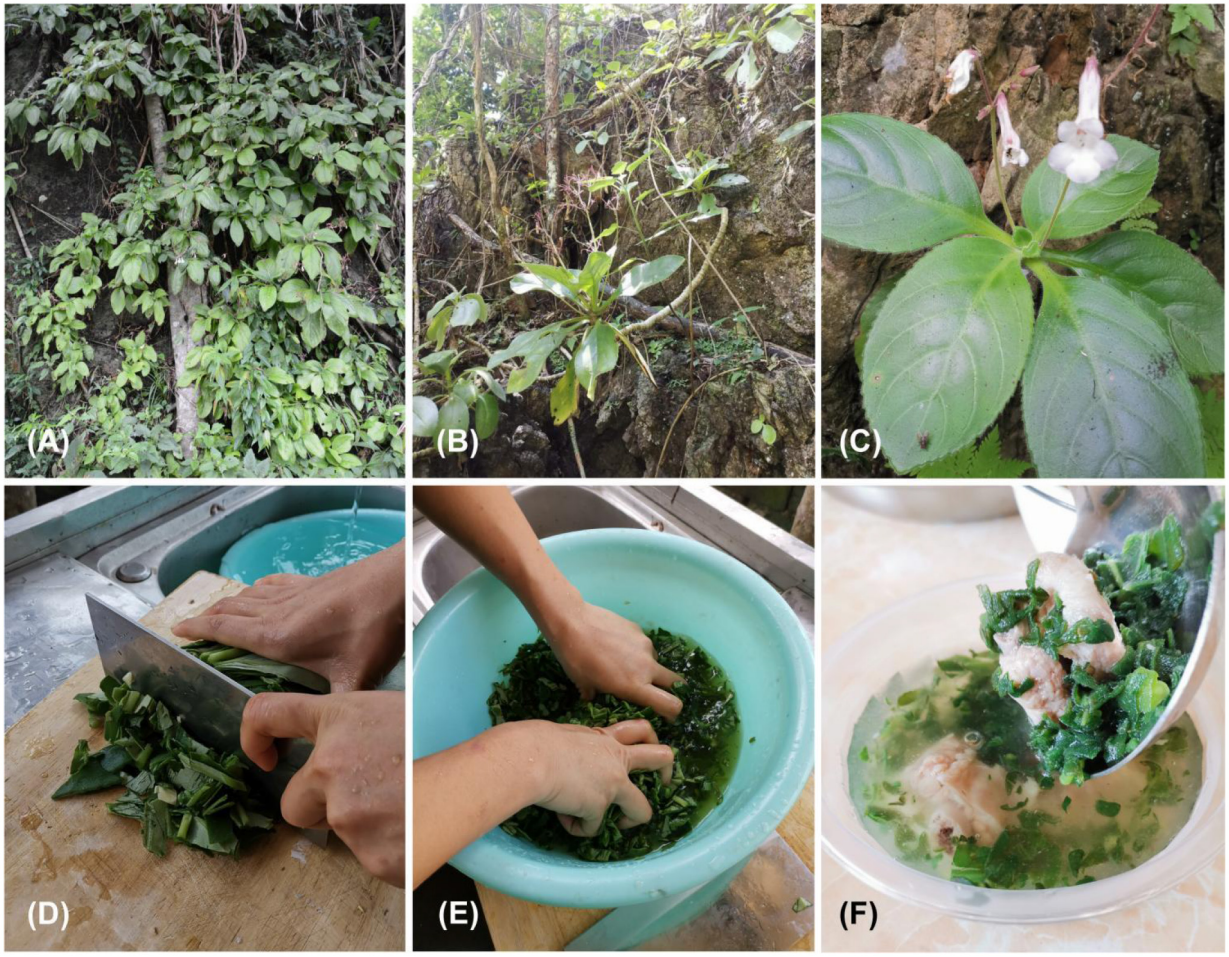
Primulina heterotricha and its traditional pork soup (“Yanye Paigu Tang”; 烟叶排骨汤) prepared by the Li ethnic group. **(A)** Habitat, **(B)** habit, **(C)** flowering individual in the wild, **(D)** leaves chopped, **(E)** rubbed, **(F)** pork soup with leaves.

The experimental samples were leaves of *P. heterotricha* collected from Bawangling (19.1°N, 109.2°E) in Changjiang Li Autonomous County, Hainan Province, China (Figure 1). We collected tender leaves from five populations of *P. heterotricha*, each approximately 1 kg. The freshly collected leaves were preserved at −80 °C until they were ready for further analysis.

### 2.3 Nutritional component tests

The leaf nutrients of *P. heterotricha* were determined by different methods, including macroscopic nutrients (protein, fat, moisture, dietary fiber, and ash), trace elements (Ca, Mg, P, K, Fe, Na, Zn, Cu, and Se), and vitamins (A, B1, B2, C, D, and E). The analysis methods and detected items are listed in Table 1. The determined methods mostly followed the Standardization Technical Guidance Document of the People's Republic of China and International Organization for Standardization (ISO).

**Table 1:** Determination of nutritional components in leaves of *Primulina heterotricha*.

**Test items**	**Determination methods**	**References**
**Macronutrient**		
Protein	GB 5009.5-2016 method 1	(72)
Fat	GB 5009.6-2016 method 2	(73)
Moisture	GB 5009.3-2016 method 1	(74)
Dietary fiber	GB 5009.88-2014	(75)
Ash	GB 5009.4-2016 method 1	(76)
Trace elements	GB 5009.268-2016 method 2	(77)
**Vitamins**		
Vitamin A	GB 5009.82-2016 method 1	(78)
Vitamin B1	GB 5009.84-2016 method 2	(79)
Vitamin B2	GB 5009.85-2016 method 1	(80)
Vitamin C	GB 5009.86-2016 method 2	(81)
Vitamin D	GB 5009.82-2016 method 1	(78)
Vitamin E	GB 5009.82-2016 method 1	(78)

The carbohydrate content was calculated as: Carbohydrate = 100 – (moisture + total ash + crude fat + crude fiber + crude protein) and the energy value was determined by multiplying the values obtained for carbohydrate, crude fat, and crude protein by 4, 9, and 4 respectively and summing up the products (23).

### 2.4 Calcium speciation tests

In the laboratory, leaf samples were initially fixed at 105 °C for 30 min, followed by drying at 80 °C for 12 h. The dried samples were subsequently ground and sieved through a 100-mesh sieve to obtain fine leaf powder for analysis. The determination of calcium speciation in the leaves was performed based on the method described by Ohat et al. (24), with minor modifications. The extraction protocol for sequentially fractionating different forms of calcium was as follows: 80% C_2_H_5_OH was first employed to extract calcium nitrate and calcium chloride (AIC-Ca); distilled water was then used to extract the water soluble calcium (H_2_O-Ca); 1 M NaCl was subsequently applied to extract calcium pectate (NaCl-Ca); 2% CH_3_COOH was used to extract calcium phosphate and calcium carbonate (HAC-Ca); finally, 0.6% HCl was used to extract calcium oxalate (HCl-Ca). After these extractions, the remaining residue contained silicate-bound calcium (Res-Ca).

For each extraction step, precisely 0.500 ± 0.0005 g of leaf powder was weighed into a 50 ml centrifuge tube with a cap. To this, 20 ml of the corresponding extractant was added, and the mixture was shaken in a thermostatic water bath at 30 °C for 1 h. The suspension was then centrifuged at 4,000 rpm for 10 min, and the resulting supernatant was filtered into a 50 ml volumetric flask. Each extraction was repeated twice more under the same conditions. The sequential extractions were performed according to the above procedure with each respective extractant. The supernatants from all extraction steps were diluted to volume with 5% hydrochloric acid and filtered through quantitative filter paper; blank samples were processed in parallel for accuracy control.

Following the completion of the five extraction steps, the residual solid was transferred to a clean tall beaker. The mixture was evaporated to dryness on a hotplate. Subsequently, 5 ml of HNO_3_-HClO_4_ (4:1, v/v) solution was added in a fume hood, thoroughly mixed, and allowed to stand overnight on a hotplate at 50 °C. On the following day, an additional 10 ml of HNO_3_-HClO_4_ (4:1, v/v) was added, and the mixture was digested at 80 °C for 30 min, 150 °C for 1 h, and then at 180 °C until the initially observed brown fumes dissipated and turned white. Once the white fumes had ceased and the liquid had evaporated completely, 15 ml of 0.2% HNO_3_ was added in two sequential portions, with heating to ensure complete dissolution of the precipitate. After cooling, the solution was quantitatively transferred to a 25 ml volumetric flask, diluted to volume with 0.2% HNO_3_, mixed thoroughly, and filtered through a 0.45 μm membrane to yield the residual calcium extract. Blank and standard samples were digested concurrently to enable quality control and correction of results. The concentrations of residual calcium and all previously extracted calcium fractions were determined by atomic absorption spectrophotometry (PinAAcle 900T, PerkinElmer).

## 3 Results

### 3.1 Ethnobotanical knowledge

Based on the literature, the Li people primarily use the leaves of *Primulina heterotricha* to prepare soup (8). They harvest the tender leaves, which are thoroughly washed, chopped, and rubbed before being added to the soup (Figure 1).

Ethnobotanical surveys revealed that *P. heterotricha* has exceptionally large leaves resembling those of tobacco; accordingly, local Li people refer to it as “Yanye” (烟叶; literally “tobacco leaf” in Chinese). In addition to being used in pork rib and chicken soups, the leaves are also consumed as a stir-fried vegetable. Notably, local people exclusively harvest tender leaves (intermediate between young expanding and senescent leaves), while leaving other plant parts intact, thereby allowing continuous use of the plant.

Furthermore, interviews with local traditional healers indicated that the fresh leaves are also used in folk medicine, where they are believed to have antihypertensive effects. Importantly, only fresh leaves are collected, and no other plant parts are used, in order to ensure normal plant growth and reproduction. This selective harvesting practice reflects a strong local awareness of sustainable plant use and conservation.

### 3.2 Nutrition components

In oder to verify the scientificity of the traditional use of *P. heterotricha*, we analyzed the contents of energy, protein, fat, moisture, carbohydrate, dietary fiber, ash, trace elements, vitamins in *P. heterotricha* leaves (results are shown in Table 2, Supplementary Table S1).

**Table 2:** Nutritional composition in leaves of *Primulina heterotricha*.

**Nutrient**	**Composition**	**Daily intake**
Energy (kcal)	3.72 ± 1.31	—
Moisture (g/100 g)	95.4 ± 0.22	—
Ash (g/100 g)	0.720 ± 0.09	—
**Macronutrient (g/100 g)**		
Protein	0.0210 ± 0.01	55–65 g
Fat	0.200 ± 0.02	770–997.5 kcal (/kg)
Carbohydrate	0.709 ± 0.34	120–150 g
Dietary fiber	2.95 ± 0.03	27 g
**Trace elements (mg/100 g)**		
Ca	289 ± 1.32	800 mg
Mg	41.5 ± 1.74	330 mg
P	14.3 ± 1.72	720 mg
K	11.0 ± 1.09	2,000 mg
Fe	1.98 ± 0.21	12–20 mg
Na	1.76 ± 0.38	1,500 mg
Mn	0.758 ± 0.07	4.5 mg
Zn	0.172 ± 0.02	7.5–12.5 mg
Cu	0.0272 ± 0.01	0.8 mg
Se	0.00210 ± 0.0004	60 μg
**Vitamins (mg/100 g)**		
Vitamin A	0.0188 ± 0.01	0.7–0.8 mg
Vitamin B1	0.149 ± 0.02	1.2–1.4 mg
Vitamin B2	0.0837 ± 0.03	1.2–1.4 mg
Vitamin C	0.104 ± 0.08	100 mg
Vitamin D	Undetected	10 μg
Vitamin E	0.0929 ± 0.01	14 mg

Values are expressed as mean ± SD, *n* = 5; Recommended daily intake amounts are based on Yang (27).

The leaves of *P. heterotricha* are characterized by a high moisture content of 95.4 ± 0.22 g/100 g, ranking second only to lettuce (96.7 g/100 g). In addition, they contain a relatively high level of dietary fiber (2.95 ± 0.03 g/100 g), slightly lower than that reported for kale (3.20 g/100 g), and a moderate fat content (0.200 ± 0.02 g/100 g; Supplementary Table S1). Furthermore, the tender leaves of *P. heterotricha* can providing 3.72 ± 1.31 kcal/100 g of energy, and their available carbohydrate content is 0.709 ± 0.34 g/100 g. In contrast, their protein content is very low (0.0210 ± 0.01 g/100 g), and the ash content is also relatively low at 0.720 ± 0.09 g/100 g.

The leaves of *P. heterotricha* are rich in several mineral elements, particularly calcium, which reaches 289 ± 1.32 mg/100 g, followed by magnesium (41.5 ± 1.74 mg/100 g), iron (1.98 ± 0.21 mg/100 g), and manganese (0.758 ± 0.07 mg/100 g). Based on the recommended daily intake for an average adult, approximately 277, 795, 606, and 594 g of leaves would be needed to meet the daily requirements for Ca, Mg, Fe, and Mn, respectively. The contents of phosphorus, potassium, sodium, zinc, copper, and selenium were comparatively moderate.

Regarding vitamins, the leaves contained a relatively high level of Vitamin B1 (0.149 ± 0.02 mg/100 g), while the levels of Vitamins A, B2, C, D, and E were comparatively low.

### 3.3 Content of various calcium speciation in leaves

**Figure 2:**
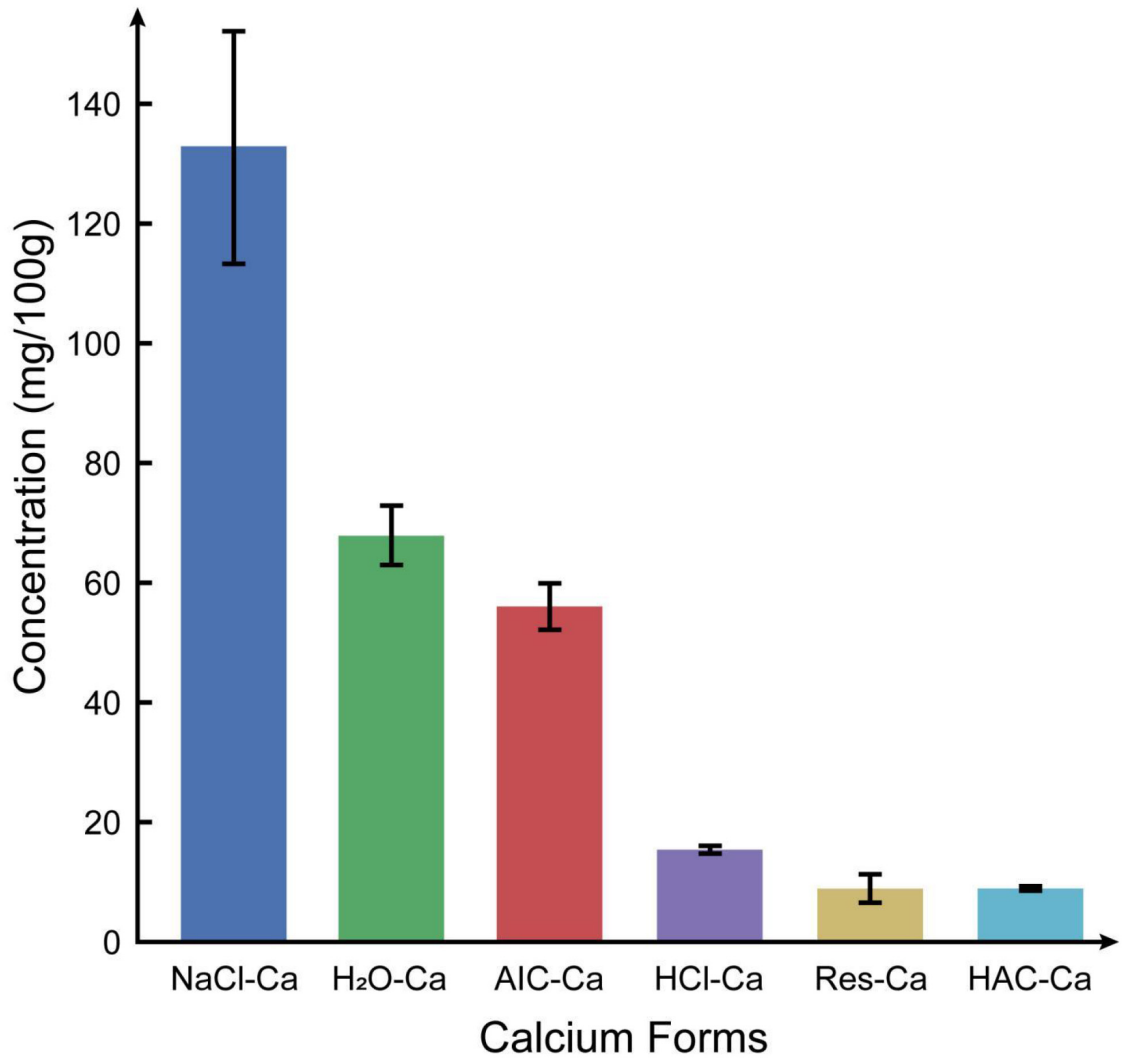
Calcium speciation and concentrations in leaves of *Primulina heterotricha*.

The forms of calcium present in the leaves of *P. heterotricha* are shown in Figure 2, with the following order: NaCl-Ca (133 ± 19.29 mg/100 g) > H_2_O-Ca (67.8 ± 4.97 mg/100 g) > AIC-Ca (55.9 ± 3.90 mg/100 g) > HCl-Ca (15.3 ± 0.82 mg/100 g) > Res-Ca (8.80 ± 2.32 mg/100 g) > HAC-Ca (8.60 ± 0.12 mg/100 g). These results indicate that the majority of calcium exists in the NaCl-Ca form.

## 4 Discussion

### 4.1 Nutritional composition of *Primulina heterotricha*

Compared with other common stem and leaf vegetables, *P. heterotricha* has the lowest protein content. Considering the essential role of protein in energy supply and physiological functions (25, 26), and the recommended daily intake of 55–65 g for adults (27), this indicates that *P. heterotricha* has not been a major protein source for the Li people. The near trace-level protein content observed in this study may reflect an ecological adaptation to nitrogen-poor karst environments. Karst soils are typically shallow, discontinuous, and characterized by low organic matter and rapid nutrient leaching (28, 29), which can result in chronically limited nitrogen availability and simplified nitrogen cycling. Under such conditions, the supply of plant-available nitrogen (e.g., nitrate and ammonium) is strongly constrained, which can limit key physiological processes involved in nitrogen assimilation, including nitrate reduction and subsequent amino acid synthesis (30, 31). Because nitrogen assimilation is directly linked to protein biosynthesis, long-term growth under nitrogen-limited karst soils is likely to result in intrinsically low leaf protein concentrations (32, 33). Even at the genomic level, plants inhabiting karst environments have been reported to possess smaller genome sizes, a pattern that has been linked to chronic nitrogen limitation in these ecosystems (34).

Aside from its low protein content, *P. heterotricha* exhibits a nutritional profile typical of low-energy leafy vegetables, characterized by high moisture content and low levels of fat and available carbohydrates. This combination results in a very low energy density, which is commonly associated with enhanced satiety and potential benefits for weight control (35, 36). The fat content aligns with the generally low-fat nature of vegetables (26), while the dietary fiber content is relatively high and exceeds that of several commonly consumed leafy vegetables, including *Amaranthus tricolor* (27), *Moringa oleifera* (37), and *Sesbania grandiflora* (38). High dietary fiber intake is widely recognized for its roles in promoting intestinal peristalsis, regulating glycemic and lipid metabolism, modulating gut microbiota, and potentially reducing the risk of metabolic and colorectal diseases (39–41). From a practical perspective, the high moisture content also implies a short shelf life and increased susceptibility to microbial spoilage (42–44).

Although the low ash content suggests relatively modest overall mineral accumulation and limited vitamin levels (45), wild edible plants remain important and low-cost sources of essential minerals, particularly for populations in mineral-deficient regions (46, 47). Minerals are indispensable for normal growth and physiological function, participating in enzymatic activity, energy metabolism, neuromuscular regulation, and oxygen transport (48). Notably, despite its relatively low ash content, *P. heterotricha* exhibits a high calcium concentration among vegetables, exceeding that of milk (107 mg/100 g) on a fresh-weight basis, highlighting its value as a natural dietary calcium source (27). In addition, the leaves contain appreciable levels of Mn, Mg, and Fe, which play critical roles in enzymatic reactions, metabolic processes, neuromuscular function, and hemoglobin synthesis (49–52).

Flavonoids, recognized as major bioactive compounds in *Primulina* (53, 54), contribute to antioxidant, antibacterial, and cardiovascular benefits, as well as influencing taste (55, 56). While this study did not quantify flavonoid content in *P. heterotricha*, information from local Li traditional healers indicates that the species possesses antihypertensive properties. These findings highlight its potential as a functional food. Future research should systematically assess its bioactive compounds, verify health benefits, and develop post-harvest preservation and palatability improvement strategies to support sustainable utilization as a traditional wild vegetable.

### 4.2 Calcium content of *Primulina heterotricha*

*P. heterotricha* contains a markedly higher calcium concentration (289 mg/100 g) than most commonly consumed vegetables and, on a fresh-weight basis, exceeds the calcium content reported for milk (27). Its calcium level is also comparable to other recognized calcium-rich wild vegetables, being lower than *Moringa oleifera* (440 mg/100 g) (37) and *Sesbania grandiflora* (666 mg/100 g) (57), but higher than that of *Amaranthus tricolor* (187 mg/100 g) (27). These results indicate that *P. heterotricha* can be regarded as a high-calcium wild vegetable in terms of total calcium content.

Beyond total calcium concentration, the nutritional value of calcium depends strongly on its chemical forms and bioavailability (58, 59). In *P. heterotricha*, calcium pectate represents the dominant form, accounting for 45.9% of total calcium, followed by water-soluble calcium (23.5%). Both forms are considered biologically active and readily absorbed by the human body (59, 60). In contrast, calcium oxalate and inorganic calcium forms together constitute a relatively small proportion of total calcium, which may reduce negative effects associated with low bioavailability (61). Compared with commonly consumed leafy vegetables such as spinach and bok choy (54, 62), *P. heterotricha* exhibits a substantially higher proportion of bioavailable calcium forms, highlighting its nutritional advantage as a calcium source.

High calcium accumulation appears to be a common characteristic of *Primulina* species from karst regions, as reported in several congeners (41, 62–65). Our results are consistent with this pattern and further confirm that the calcium content of *P. heterotricha* is 2–21 times higher than that of common leafy vegetables (54, 62). Notably, *P. heterotricha* is endemic to Hainan Island and has long been traditionally consumed by the local Li ethnic group, suggesting that this high-calcium trait has practical dietary relevance. Together, these findings indicate that *P. heterotricha* represents a locally important and nutritionally valuable calcium-rich wild vegetable.

### 4.3 Traditional knowledge under social change

The Li ethnic group, a minority uniquely distributed on Hainan Island that transitioned directly from traditional to contemporary transitions (6), possesses its own spoken language but lacks a writing system, relying entirely on oral tradition for the transmission of knowledge. Most Li people live in remote mountainous areas, maintaining a small-scale, self-sufficient agrarian economy (66). Surrounded by dense vegetation, plants form an essential part of their diet and daily life (67). Over generations, the Li have accumulated extensive ethnobotanical knowledge, encompassing wild edible plants, medicinal species, and culturally significant flora, which is traditionally transmitted orally from elders to younger generations.

However, rapid modernization and urban migration among younger Li are leading to a growing detachment from ancestral environments and traditional livelihoods. Consequently, knowledge and skills related to identifying, gathering, and utilizing wild plants—particularly endemic species with narrow distributions such as *P. heterotricha*—are at risk of being lost (66, 68). Detailed knowledge of these species is often held by only a few elders, making its preservation urgent.

Under the “big food concept,” which emphasizes diversification of food sources and the sustainable use of wild edible and medicinal plants, traditional ecological knowledge has gained renewed attention (69, 70). The development of Li society reflects extensive plant use, with a cultural ethos emphasizing harmony with nature, environmental respect, and resource conservation (66). In social-ecological systems, biological and cultural diversity are closely intertwined (71); thus, preserving traditional knowledge contributes simultaneously to conserving both plant biodiversity and cultural heritage.

## 5 Conclusion

This study systematically analyzed the ethnobotanical origins and nutritional value of *Primulina heterotricha*, a unique wild vegetable traditionally consumed by the Li ethnic group of Hainan Island. The results demonstrate that this plant, as a nutrient-rich traditional food, holds promise as a distinctive ingredient with both nutritional and culinary potential, pending further verification of its safety and bioactive compounds. The development of such specialty vegetables may represent a promising candidate for future inclusion in sustainable local food systems. However, the culinary value and unique characteristics of these non-conventional vegetables are currently known only in limited regions, highlighting the urgent need for targeted promotion to disseminate knowledge, increase product awareness, and stimulate consumer interest. For leafy vegetables like *P. heterotricha*, future breeding or cultivation efforts should prioritize enhancements in dietary fiber, vitamins, protein content, disease resistance, and palatability. Such measures will support the conservation and sustainable utilization of this valuable yet underutilized plant resource.

## Data Availability

The original contributions presented in the study are included in the article/Supplementary material, further inquiries can be directed to the corresponding authors.
